# Prelude to Intersex in Fish: Identifying a Sensitive Period for Feminization

**Published:** 2005-10

**Authors:** Angela Spivey

Field studies have shown a high occurrence of intersex (the presence of both male and female characteristics) and ovotestis (the presence of eggs in the testis) in wild populations of a fish known as roach (*Rutilus rutilus*) in rivers in the United Kingdom that are downstream from wastewater treatment plants. Furthermore, studies have demonstrated that intersex males are less fertile, which may have population-level effects. However, to date, scientists have been unable to induce intersex in male fish with controlled exposures to wastewater effluents. A study conducted at The University of Exeter now shows that the sensitive period for feminization of the reproductive duct—in which the male testis forms an ovary-like cavity—may occur earlier than previously thought, and raises new questions about the conditions that lead to actual germ cell disruption [***EHP* 113:1299–1307]**.

Many questions persist about the causes of and the most vulnerable life stages for various types of sexual effects induced by estrogenic chemicals in wastewater effluent. In this study, the researchers collected two different U.K. wastewater effluents and exposed wild roach at two life stages: during early life and development of the gonads (from fertilization up to 300 days post-hatch) and as adults producing germ cells following annual spawning. These adults included one group of fish that had been raised in clean water and another that had hatched and grown to maturity in the wild.

Both effluents induced synthesis of vitellogenin (an estrogen-dependent yolk precursor and biomarker of estrogen exposure) at both life stages, with the extent of this induction correlating with the steroid estrogen content of the effluent. Previous studies have demonstrated that feminization of the sperm duct to form an ovary-like cavity occurs when exposure to effluent comes during the time of sexual differentiation, which in roach occurs from 50 to 150 days post-hatch. This study showed alteration of the sperm duct with an exposure earlier in life, from fertilization to 60 days post-hatch, before any signs of sexual development appear. The alteration, furthermore, was permanent, persisting even after 240 days’ maintenance in clean water after exposure.

However, no ovotestis was observed in any of the juvenile fish. There was also no evidence of ovotestis in post-spawning adult male roach raised in a clean environment and subsequently exposed to effluent. There was evidence that the wild males had previously been exposed to estrogenic stimuli, as some of males had ovotestis when the study began. The severity of this condition increased slightly during the study period, but the increase occurred across both exposed and control fish and thus appeared unrelated to the study effluent exposure.

The authors suggest possible explanations that need further study—one is that ovotestis is induced only by effluents with greater levels of estrogenic chemicals than those used in the study. The researchers evaluated the effluents for content of two chemicals previously implicated in causing intersex—steroidal estrogens and alkylphenols—and found that these levels were similar to concentrations reported in wastewater effluents in the United Kingdom and worldwide. They emphasize that chemical content and interactions ideally should be taken into account when trying to determine the conditions that lead to sexual effects.

The results of these studies raise the possibility that ovotestis may be a result either of longevity of exposure or of programming in early life that manifests itself as fish mature sexually. Previous findings from the authors support this idea by showing that the severity of intersex increases with age. The authors are further exploring these possibilities now with a laboratory study of roach that includes an environmentally relevant estrogen exposure of two years’ duration.

